# Greenhouse Gas Emissions Accounting of Urban Residential Consumption: A Household Survey Based Approach

**DOI:** 10.1371/journal.pone.0055642

**Published:** 2013-02-06

**Authors:** Tao Lin, Yunjun Yu, Xuemei Bai, Ling Feng, Jin Wang

**Affiliations:** 1 Key Lab of Urban Environment and Health, Institute of Urban Environment, Chinese Academy of Sciences, Xiamen, China; 2 Xiamen Key Lab of Urban Metabolism, Xiamen, China; 3 Fenner School of Environment and Society, Australian National University, Canberra, Australia; MIT, United States of America

## Abstract

Devising policies for a low carbon city requires a careful understanding of the characteristics of urban residential lifestyle and consumption. The production-based accounting approach based on top-down statistical data has a limited ability to reflect the total greenhouse gas (GHG) emissions from residential consumption. In this paper, we present a survey-based GHG emissions accounting methodology for urban residential consumption, and apply it in Xiamen City, a rapidly urbanizing coastal city in southeast China. Based on this, the main influencing factors determining residential GHG emissions at the household and community scale are identified, and the typical profiles of low, medium and high GHG emission households and communities are identified. Up to 70% of household GHG emissions are from regional and national activities that support household consumption including the supply of energy and building materials, while 17% are from urban level basic services and supplies such as sewage treatment and solid waste management, and only 13% are direct emissions from household consumption. Housing area and household size are the two main factors determining GHG emissions from residential consumption at the household scale, while average housing area and building height were the main factors at the community scale. Our results show a large disparity in GHG emissions profiles among different households, with high GHG emissions households emitting about five times more than low GHG emissions households. Emissions from high GHG emissions communities are about twice as high as from low GHG emissions communities. Our findings can contribute to better tailored and targeted policies aimed at reducing household GHG emissions, and developing low GHG emissions residential communities in China.

## Introduction

More than half of the world’s population are living in cities and urbanization is transforming the global environment at unparalleled rates and scales [1], [2]. Cities are estimated to account for about 78% of total global greenhouse gas (GHG) emissions, but are also the loci for innovative solutions to reduce emissions [3]–[8]. Household lifestyle has been recognized as a major driver of energy use and related GHG emissions besides technology efficiency [9]–[14]. Carbon management in cities is increasingly focusing on individuals, households, and communities due to population growth and improved living standards of urban residents [14]–[19]. A better understanding of urban residential consumption patterns in relation to urban system structure and processes, and their linkages to GHG emissions emission profiles, will enable cities to develop tailor-made planning and policy measures towards low carbon cities.

The present accounting methods of GHG emissions can be roughly categorized into production-based and consumption-based accounting approaches [20], [21]. Production-based approaches are always exemplified in national-scale inventories and tracks mainly the direct GHG emissions across all production sectors and the residential sector within the political or geographical boundary [20], [22]. These approches do not include energy embodied in imported goods and services. Strict boundary-limited GHG accounting is unsuitable for cities because they don’t include embodied emissions in imported goods and services. Theoretically, consumption-based accounting provides the most rigorous GHG estimation incorporating transboundary emissions. Consumption-based approaches link the consumption levels and patterns of urban residents with the associated direct and embodied GHG emissions whether those occur inside or outside the city boundary, through the proxy of local household expenditure. As a result, in cities with significant export-related industrial activities and relatively low resident populations, the consumption-based accounting approach will likely lead to lower GHG emissions estimates compared to production-based accounting approaches. Conversely, for residence and service-oriented cities that typically import all energy and energy-intensive materials and goods, consumption-based accounting approach will more likely yield substantially higher estimation compared to production-based accounting approaches [22]. Production-based accounting approaches often based on top-down statistical data which uses same categories and definitions and is internally consistent to allow comparisons and benchmarking. While consumption-based accounting approaches are always based on an extensive city wide survey and only a limited number of consumption-based accounts for cities are available [23]. Sampling errors in consumption surveys may add some degree of uncertainty [24]. However, it can reflect consumption choices and empower households and governments to redirect a low-carbon lifestyle [20].

The last three decades have seen unprecedented urbanization in China, from 19% in 1980 to 51% in 2011, and this rapid urbanization is expected to continue in the coming decades. Currently, the 35 largest cities contain 18% of the national population, but account for 40% of China’s energy use and GHG emissions [25]. The socioeconomic development in Chinese cities and large numbers of new urban migrants has driven significant increases in energy use and related GHG emissions, because urban communities have a greater per capita energy demand than rural settlements [26]. Changing urban lifestyles will play an increasingly important role in shaping China’s energy demand and GHG emissions. However, existing research on GHG emissions accounting in China mostly employ production-based accounting using top-down government statistics, and embodied energy use and GHG emissions driven by residential consumption are often omitted or underestimated.

In this paper, we present a survey based GHG emissions accounting methodology for urban residential consumption, and apply it in Xiamen City, a rapidly urbanizing coastal city in southeast China. Based on our results, we explore the current main influencing factors determining residential GHG emissions at the household and community scale, and present typical profiles of low and high GHG emission households and communities. Based on the results, policy implications for developing a low GHG emissions urban consumption pattern are discussed.

## Methods

Our study consists of four steps: (1) designing a city-wide questionnaire survey; (2) defining the system boundary, establishing consumption categories and GHG emissions accounting methodology for seven consumption categories; (3) conducting the survey; and (4) data processing and analysis of the survey results, including influencing factor analysis and profiling of low, medium and high GHG emission households and communities. Our study obtained ethical approval from the Academic Committee of the Institute of Urban Environment (IUE), Chinese Academy of Sciences.

### Survey Design

In our study, all the data for GHG emissions accounting of urban residential consumption and influencing factor analysis are derived from an onsite questionnaire survey. The questionnaire consists of two components: household information and residential consumption. The survey variables of each component are listed in Table 1. GHG emissions accounting of urban residential consumption focuses on seven categories including electricity use, fuel consumption, transportation, solid waste treatment, wastewater treatment, food, and housing (which is treated as a consumable durable good). The quantity consumed in each category was collected directly or converted from the surveyed residential consumption variables, for example, we calculated the actual water consumption by dividing the surveyed water rate of household by current water price. The influencing factors of urban residential GHG emissions in our study were classified into variables at household and community scale. Residential status (permanent population or transient population), marital status, household size, age, household income, housing area, education, building age, and number of houses were considered to be potential influencing factors at the household scale. Average housing area, building age, average household income, building height, and average household size were considered to be potential influencing factors at the community scale.

**Table 1:** Components and survey variables in residential consumption questionnaire.

Components	Survey variables
Household information	Residential status; marital status; household size; age; education;
	household income
Residential consumption	Number of houses; housing area; building Height; building age;
	water fee; power fee; gas fee; waste production; food
	consumption; transportation destination; mode of transport; trip
	frequency; travel time

In view of the heterogenous spatial demographics of households and residential communities, we applied the spatial sampling method, which takes the spatial distribution characteristics of the object into account. The principle of this method is to balance the cost of sampling with the desired sampling precision, depending on study objectives and spatial variation [27], [28]. The spatial distribution characteristics in our study included topography, population density, standard land price, and administrative division.

### System Boundary and Accounting Methodology

**Figure 1:**
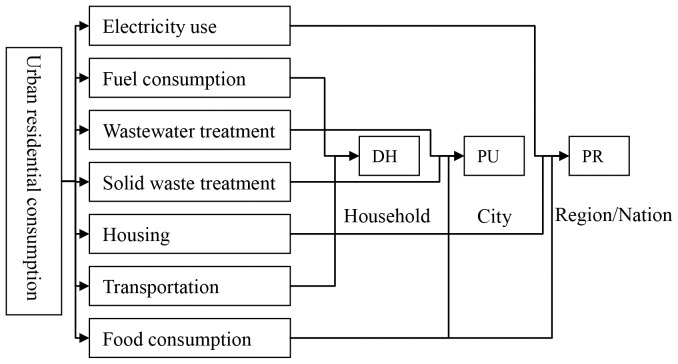
Description of system boundary of accounting methodology. Note: GHG emissions from food consumption was partially PU-sourced, since about one-third of food consumption in Xiamen is self-supplied.

In our study, the GHG emissions accounting of urban residential consumption was classified into seven categories: housing, electricity use, fuel consumption, wastewater treatment, solid waste treatment, transportation and food consumption. Those residential consumptions had covered the key urban infrastructural flows and materials [20], [29]. As for the data collection limited, the embodied emissions in manufactured goods, appliances and water supply were left out. GHG emissions were expressed in carbon dioxide equivalents (CO_2_e) and different greenhouse gases (GHGs) were converted into CO_2_e emissions by using IPCC global warming potential (GWP) parameters [30]. The system boundaries varies according to different categories of residential consumption. The seven GHG emission categories were therefore classified into three sources according to the general path of primary energy or materials to the end-users [22]: primary equivalent GHG emissions from regional and national economic activity supplied to meet household demand (referred to as PR-sourced hereafter), primary equivalent GHG emissions from urban economic activities supplied to household demand (PU-sourced), and household direct GHG emissions from household activities (DH-sourced). Figure 1 shows the spatial extension of the system boundaries for each of the seven categories.

#### 1. GHG emissions from electricity use and fuel consumption

GHG emissions from electricity use and fuel consumption were derived from the direct energy use of household activities such as cooking, heating and lighting, and household appliances such as computer, television and refrigerator. GHG emissions accounting of these two consumption categories commonly multiply the actually consumed amount by the corresponding emission factors. The GHG emissions from electricity use and fuel consumption are respectively calculated using the following two formulas:
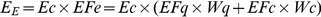
(1)Where, *E_E_* is GHG emissions from residential electricity per month; *E*_c_ is amount of residential electricity consumption per month; *EF*_e_ is the emission factor of electricity. *EF*_q_ and *EF*_c_ are the marginal emission factor of electrical quantity and marginal emission factor of electrical capacity of the East China Power Grid in 2009, which represent the the emission factors of currently running plants and newly built plants charged by East China Power Grid respectively [31]; *W*_q_ and *W*_c_ are respective weights of the emission factors for electricity. Here, we assign the same value to the two weights.
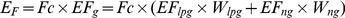
(2)Where, EF is GHG emissions from residential gas consumption per month; Fc is amount of residential gas consumption per month; EFg is the emission factor of gas. EFlpg is emission factor of liquefied petroleum gas; EFng is emission factor of natural gas; Wlpg, Wng are weights of the emission factors for liquefied petroleum gas and natural gas respectively. Here, we assign the values to the two weights according to the gas consumption proportion in Xiamen City (0.63 for liquefied petroleum gas and 0.37 for natural gas). The emission factors are referenced from the 2006 IPCC guidelines for national greenhouse gas inventories [30].

#### 2. GHG emissions from transportation

The GHG emissions from transportation were estimated according to different modes of transport and corresponding consumption of diesel, petrol, gas or electricity. According to the GHG emissions accounting method for mobile sources [30], we calculate the GHG emissions by multiplying GHG emissions intensity per unit time with the travel time of each travel mode, according to the following two formulas:
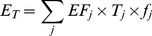
(3)Where *E_T_* is total GHG emissions from transportation per month. *EF*_j_ is emission factor per unit time of travel mode *j*; *T_j_* is average travel time of travel mode *j*; Travel mode *j* represents walking, cycling, private car, taxi, public bus, bus rapid transit (BRT), shuttle bus, or motorcycle respectively. *f_j_* is frequency of travel mode *j*. The *EF* of walking and biking is 0; the motorcycle *EF* is estimated through electricity consumption per unit time, as most motorcycles in Xiamen City are electric powered. The *EF* of private car, taxi, public bus, BRT, and shuttle bus are estimated as follows:
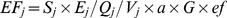
(4)Where Sj is total operation mileage per unit time of mode j; Ej is fuel consumption per unit distance; Qj is passenger volume per unit time of travel mode j; Vj is travel time per capita of travel mode j; a is fuel density of diesel or gasoline; G is net heat value of diesel or gasoline; ef is emission factor of diesel or gasoline (see Table 2).

**Table 2:** Parameters for estimating the emission factors of different travel modes in Xiamen City.

Travel mode	*S_j_* a,d	*E_j_* a	*Q_j_* a	*V_j_* a	Fuel type	Calorific value ^b^	EF^ c^
	(100km/a)	(L/100km)	(P/a)	(minute)		(kJ/kg)	(tC/TJ)
Taxi	62,055,780	10.5	22,813	25.46	gasoline	43,124	69,2
Bus	1,763,045	25	41,180	25.46	diesel	42,705	74,0
BRT	26,825	36	2,375	25.46	diesel	42,705	74,0
Shuttle	536,954	23	15,243	25.46	diesel	42,705	74,0

Notes:

a The data of Sj, Ej, Qj and Vj are derived from Xiamen City’s Transportation Committee and Xiamen Transportation Company. ^b^ Calorific values are taken from ‘General calculation principles for total production energy consumption (GB/T-2589–2008)’ (in Chinese). ^c^ Emission factors were extracted from the Technology and Environmental Database (TED) in Lin’s study [6]. ^d^ This equation will always underestimate the total emissions due to transport because the parameter S_j_ does not record fuel use while a vehicle is stationary.

#### 3. GHG emissions from food consumption

The GHG emissions from food consumption mainly consist of direct emissions from human metabolism and indirect emissions from food processing and supply. As the direct GHG emissions from food consumption by human metabolism will overlap the GHG emissions from wastewater treatment, here only the indirect GHG emissions from food processing and supply are calculated using the following formulas:
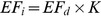
(5)Where *EF_i_* is indirect GHG emissions from food consumption; *EF_d_* is direct GHG emissions from food consumption; *K* is proportion of *EF_i_* to *EF_d_* and refers to the proportion of indirect GHG emissions to direct GHG emissions from Chinese residential food consumption in 2006 [32]. *EF_d_* is estimated as follows:
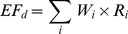
(6)

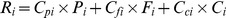
(7)Where *W_i_* is consumption amount of food *i*; *R*_i_ is carbon content of food *i*; *C_p_*_i_, *C_fi_*, and *C_ci_* are contents of protein, fat, and carbohydrate of food *i* respectively; *P_i_*, *F_i_*, and *C_i_* are the carbon content of protein, fat, and carbohydrate respectively; *C_pi_*, *C_f_*_i_, and *C_ci_* refers to *China food composition* [33].

#### 4. GHG emissions from household solid waste treatment

In 2009, household solid waste disposal and treatment in Xiamen City included landfill (80%) and incineration (20%) The GHG emissions from landfill diposal mainly considered to be emissions of CH_4_ and CO_2_ from the landfill yard, which can be estimated as follows [34]:
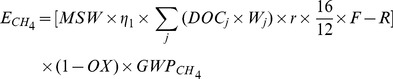
(8)

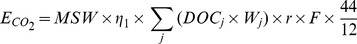
(9)Where *E_CH4_* and *E_CO2_* are amount of CH_4_ and CO_2_ emitted from solid waste disposal respectively; *MSW* is mass of solid waste deposited in Xiamen City in 2009; *η_1_* is proportion of solid waste deposited to landfill; *DOC_j_* is fraction of degradable organic carbon to degradable component *j*; *W_j_* is fraction of degradable component *j* to total solid waste deposited; 16/12 is molecular weight ratio CH_4_/C; *r* is fraction of degradable organic carbon that can decompose; *F* is volume fraction of CH_4_ in generated landfill gas; *R* is the recovery rate of CH_4_; *OX* is the oxidation rate of CH_4_; *GWP_CH4_* is the global warming potential of CH_4_.

According to *2006 IPCC guidelines for national greenhouse gas inventories* [30], the GHG emissions from landfill incineration is mainly from CO_2_ and N_2_O and can be estimated as follows:
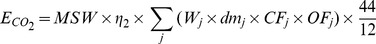
(10)

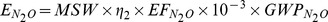
(11)Where *E_CO2_* and *E_N2O_* are amount of CO_2_ and N_2_O emitted from solid waste incineration; *η_2_* is proportion of solid waste deposited by incineration; *dm_j_* is dry matter content of degradable component *j; CF_j_* is fraction of carbon in degradable component *j*; *OF_j_* is oxidation factor; 44/12 is the molecular weight ratio CO_2_/C; *EF_N2O_* is emission factor of N_2_O from waste incineration; *GWP_N2O_* is global warming potential of N_2_O.

#### 5. GHG emissions from household wastewater treatment

In our study, all the household water used was assumed to be transformed to wastewater. The calculation of GHG emissions from wastewater treatment mainly considered sewage plant emissions of CH_4_ which are produced from anaerobic treatment process and can be calculated as follows:
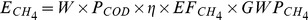
(12)Where *E_CH4_* is production of CH_4_ from wastewater treatment; *W* is mass of wastewater; *P_COD_* is content of chemical oxygen demand in wastewater; *η* is fraction of wastewater through anaerobic treatment; *EF_CH4_* is emission factor of CH_4_.

#### 6. GHG emissions from housing

In our study, housing is considered to be a durable consumable good with a lifetime of fifty years, as this is the maximum service life of residential housing regulated by the Ministry of Housing and Urban-Rural Development, PR China. Lifecycle GHG emissions result from material mining and processing, construction, house operation and demolition, but material mining and processing and house operation are responsible for most of the emissions. In principle, GHG emissions from housing operation should be the same to those from household electricity use and fuel consumption, so the GHG emissions from housing mainly considered lifecycle GHG emissions from building materials. There are two types of residential buildings (masonry-concrete and steel-concrete) in Xiamen. Liu et al. estimated the energy consumption and environmental emissions of the two types of residential building using life cycle analysis and the Boustead Model [35]. Based on her estimation of GHG emissions per unit area for the two types of residential building (see Table 3), GHG emissions from the two types of housing can be calculated as follows:
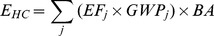
(13)Where *E_HC_* is GHG emissions from housing; *EF_j_* is emission amount of greenhouse gas *j*; *j* represents CO_2,_ CO, and N_2_O respectively; *GWP_j_* is global warming potential of gas *j*; *BA* is the building area.

**Table 3:** GHG emissions per unit area in the lifecycle of building materials.

GHGs	GHG emissions in the lifecycle kg/m^2^		*GWP*_j_
	Steel-concrete a	Masonry-concrete a	
CO	20.1	7.5	2
CO_2_	954.2	828.51	1
NO_x_	6.2	2.68	310

Note:

a the emission factors of steel-concrete and masonry-concrete refer to Liu’s study [33].

### Study Area and Survey Implementation

Xiamen is a typical coastal city located in southeast China (24°25′N-24°55′N, 117°53′E-118°27′E). It has a land area of 1,565 km^2^ and a sea area of 390 km^2^ [36]. The rapid urban expansion and economic development of Xiamen was not triggered until the implementation of China’s ‘reform and opening-up’ policy in 1980, when the Xiamen Special Economic Zone was established on the island. Since then, Xiamen has undergone rapid urbanization and its urban population has grown at an remarkable speed. Its regional GDP reached 173.72 billion yuan in 2009, having been just 1.72 billion yuan (comparable GDP value) in 1980. Meanwhile the urbanization ratio increased rapidly from 35% to 80%, and in 2009 the population of Xiamen reached 2.52 million with a population density of 1,602 people per km^2^. Average urban disposable income and consumption expenditure reached 26,131 yuan and 17,990 yuan respectively. Residential electricity consumption was 2.75 billion kWh in 2009, up from 0.64 billion kWh in 1999. Residential water use was 9900 million ton, compared to 8300 million ton in 1999. In 2009, Xiamen became one of the first ten pilot cities of the ‘COOLCHINA-2009 civil low-carbon action pilot project’. Understanding the characteristics of GHG emissions from urban residential consumption is in urgent needed to reduce residential GHG emissions and develop a low carbon city.

**Figure 2:**
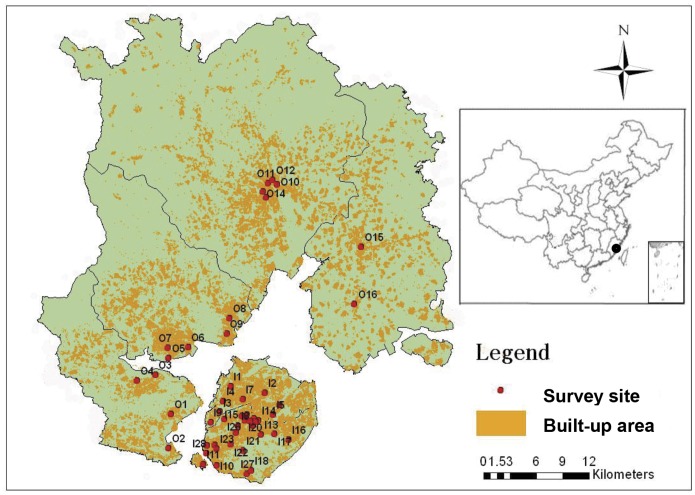
Location of Xiamen City and survey site selection.

The downtown area is located in Xiamen Island and off island districts are mainly peri-urban areas. According to the spatial sampling, 44 typical communities, including 28 from Xiamen Island (I1-I28) and 16 from Xiamen mainland (O1-O16), were determined as the survey sites (see Figure 2). The onsite questionnaire surveys were conducted in the targeted communities in October 2009 and July 2010. 1,485 questionnaires were completed, of which 714 questionnaires satisfied all the information needed in this study. This represented a sampling of about 0.1% of the total households in the targetted area.

### Data Processing and Statistical Analysis

Some questionnaire variables are quantitative (e.g. household size, water use per month) while other variables are qualitative (e.g. residential status, marriage, and education). However, all qualitative variables were transformed into ordinal variables to facilitate statistical analysis in SPSS (IBM Corporation). The transformation standards are shown in Table 4. Analysis of variance (ANOVA) which is able to test whether data from several groups have a common mean, was applied to test which potential factors would cause a significant difference (P*<*0.05) in urban residential GHG emissions. Second, a stepwise linear regression analysis was applied to identify the major influencing factors, taking the potential factors as independent variables and urban residential GHG emissions as dependent variables. Finally, taking the main influencing factors identified from regression analysis as the analysis variables, the 714 households and 44 communities of Xiamen City were respectively clustered into three GHG emission categories through K-means cluster analysis. This allowed the characteristics of low, medium and high GHG emission households and communities to be summarized and compared.

**Table 4:** Standards to transform qualitative variables into ordinal variables.

Qualitative variables	Transform standards
Residential status	Registered resident = 1; Non-registered resident = 2
Marital status	Unmarried = 1; Married = 2; Divorced = 3
Age	<25 = 1; 25∼30 = 2; 31∼40 = 3; 41∼50 = 4; 51∼59 = 5; >59 = 6
Education	Elementary = 1; Junior = 2; Senior = 3; College = 4; Graduate = 5; Others = 6
Household income	<2,000 = 1; 2,000∼5,000 = 2; 5,000∼10,000 = 3;
(yuan/month)	10,000∼20,000 = 4; >20,000 = 5
Housing area m^2^	<40 = 1; 40∼69 = 2; 70∼89 = 3; 90∼119 = 4; 120∼149 = 5; >149 = 6
Number of houses	None = 1; 1 house = 2; 2 houses = 3; >2 houses = 4
Building age	Before 1980s = 1; 1980–1990 = 2; 1990–2000 = 3; After 2000 = 4
Building Height	1–7 = 1(low-rise building); >7 = 2(high-rise building)

## Results

### GHG Emissions from Urban Residential Consumption

**Figure 3:**
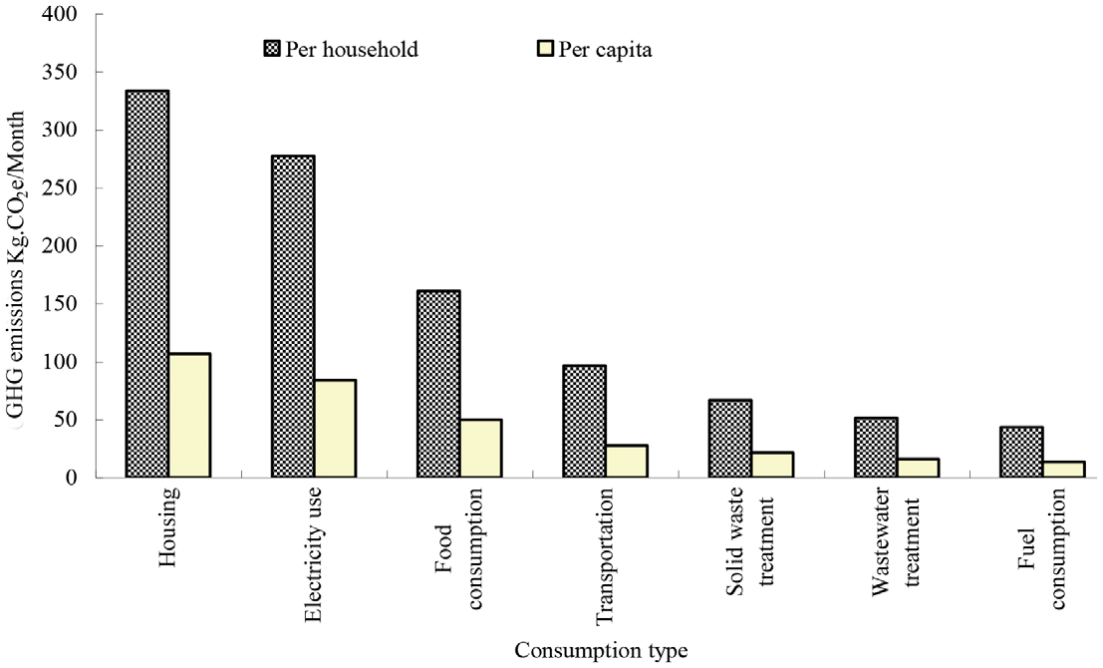
GHG emissions from residential consumption in Xiamen.

In 2009, the average GHG emissions of urban residential consumption per household in Xiamen City were 1042.31 kg CO_2_e/month. The emission intensities per household of the seven categories of residential consumption activities could be ranked in decreasing order as: housing (32.98%)>electricity use (26.84%)>food (15.17%)>transportation (9.21%)>solid waste treatment (6.44%)>wastewater treatment (5.20%)>fuel consumption (4.16%). The average per capita GHG emissions from Xiamen urban residential consumption were 323.37 kg CO_2_e/month. The order of the emission intensities per capita of the seven categories of residential consumption activities was same as for households: housing (34.11%)>electricity use (26.17%)>food (15.23%)>transportation (8.51%)>solid waste treatment (6.61%)>wastewater treatment (5.17%)>fuel consumption (4.20%) (see figure 3).

According to the system boundary classification, the majority of the GHG emissions from urban residential consumption in Xiamen City were derived from national or regional energy and material supply (PR-sourced), including building materials, electricity, and most food, which accounted for 70.43% of total GHG emissions. Urban economic activities supporting residential consumption (PU-sourced), including waste water treatment, solid waste treatment and a small fraction of food supply, accounted for 16.86% of total GHG emissions. The direct household GHG emissions (DH-sourced) only accounted for 12.71% of the total GHG emissions.

**Figure 4:**
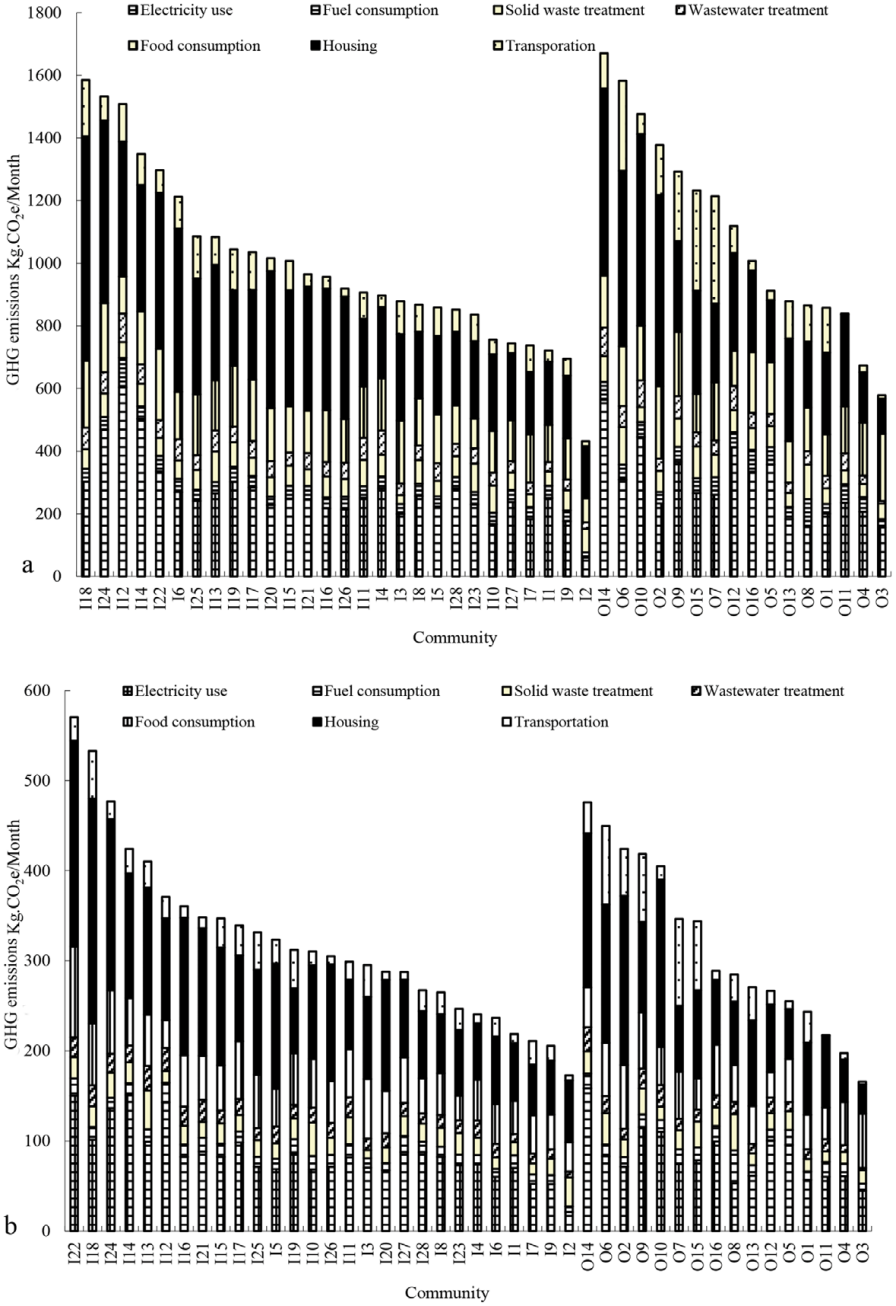
GHG emissions from residential consumptions in different communities in Xiamen City. Note: a represents GHG emissions per household; b represents GHG emissions per capita. I1-I28 represents 28 communites from Xiamen Island and O1-O16 represents 16 communities from Xiamen mainland.

At the household scale, the per household and per capita average GHG emissions from urban residential consumption of Xiamen island (downtown) communities were 991.78 kg CO_2_e/month and 321.21 kg CO_2_e/month respectively. The per household and per capita average GHG emissions from urban residential consumption per household and per capita of Xiamen mainland communities (peri-urban areas) were 1098.32 kg CO_2_e/month and 335.54 kg CO_2_e/month respectively (see figure 4). The per household GHG emissions of the downtown communities were not significantly different from of the peri-urban communities (P = 0.243). However, the per capita GHG emissions of the downtown communities were significantly lower than of the peri-urban communities (P = 0.031). The major difference between the downtown and peri-urban communities were in household electricity use and transportation. In addition, differences in average household size meant that the communities with the highest and lowest GHG emission per household were not the same as the communities with the highest and lowest GHG emissions per capita. For example, the community I18 had the highest per household GHG emissions in the downtown but its per captia GHG emissions were lower than I22 because the latter have a smaller average houshold size.

### Influencing Factors of Urban Residential GHG Emissions

Analysis of variance (ANOVA) was applied to test each survey variable to see whether it caused a significant difference (P<0.05) in total GHG emissions per household and per capita. If it did, then ANOVA was further used to test which consumption category showed a significant difference corresponding to the survey variable. The results are shown in Table 5. At the household scale, residential status, marital status, household income, housing area, education, age, building age, and number of houses can affect per household GHG emissions. Residential status, household size, household income, housing area, education, age, building age and number of houses can affect GHG emissions per capita. At the community scale, average housing area, building age, building Height and average household income can affect GHG emissions per household and per capita.

**Table 5:** One-way ANOVA analysis of potential influencing factors.

Survey variables	Total GHG emissions		Consumption categories	
	Per household	Per capita	Per household	Per capita
Residential status a	Yes	Yes	4,5,6	2,6
Marital status a	Yes	No	4,5,7	4,5,7
Household size a	/	Yes	/	1,2,3,4,5,6
Household income a	Yes	Yes	1,4,5,6,7	1,5,6,7
Housing area a	Yes	Yes	1,2,4,5,6,7	1,2,4,5,6,7
Education a	Yes	Yes	1,3,5,6,7	1,3,5,6,7
Age a	Yes	Yes	1,5,6,7	1,5,6,7
Building age a	Yes	Yes	1,2,4,5,6	1,2,4,5,6
Number of houses a	Yes	Yes	1,4,5,6,7	1,4,5,6,7
Average housing area ^b^	Yes	Yes	1,4,6,7	1,4,6,7
Building age ^b^	Yes	Yes	1,4,6	1,4,6
Building Height ^b^	Yes	Yes	1,4,5,6	1,4,5,6
Average household income ^b^	Yes	Yes	4,5,6	4,5,6
Average household size ^b^	/	No	/	4

Notes:

a represents the variables at household scale and b represents variables at community scale.

Yes means the survey variable caused a significant difference in total GHG emissions and No means not significant.

Numbers 1–7 respectively represent GHG emissions from the following seven residential consumption categories: Electricity use, Fuel consumption, Solid waste treatment, Wastewater treatment, Food consumption, Housing, and Transportation.

The results of regression analysis are shown in Table 6. At the household scale, housing area, household income, building age, household size, marital status, and age present in the regression formula of GHG emissions per household, indicating they are the influencing factors of GHG emissions per household in the statistical sense. Housing area is the main influencing factor with the largest standard regression coefficient of 0.475. Household size, housing area, building age, household income, and residential status present in the regression formula of GHG emissions per capita. Household size and housing area are the main influencing factors, with standard regression coefficients (the relative importance of the independent variables to the dependent variable) of −0.479 and 0.456 respectively. At the community scale, average housing area and building story present in both the regression formulas of GHG emissions per household and per capita. Their standard regression coefficients are respectively 0.519 and 0.497 per household and 0.455 and 0.656 per capita.

**Table 6:** Stepwise linear regression of the potential influence factors.

Independent	Unstandardized	Standardized	Independent	Unstandardized	Standardized
variables	Coefficients	coefficients	variables	coefficients	coefficients
Household scale:	per household		Household scale:	per capita	
Constant	−457.746	/	Constant	205.982	/
Housing area	201.671	0.475	Household size	−81.058	-0.479
Household income	97.823	0.178	Housing area	67.961	0.456
Household size	68.934	0.143	Building age	29.499	0.127
Building age	76.693	0.116	Household income	25.329	0.131
Marital status	130.792	0.101	Residential status	24.666	0.061
Age	−31.109	−0.072			
R^2^	0.650		R^2^	0.669	
F	84.470		F	126.068	
P	<0.001		P	<0.001	
Community scale:	per household		Community scale:	per capita	
Constant	122.132	/	Constant	28.502	/
Average housing area	226.844	0.519	Building Height	107.818	0.565
Building Height	294.515	0.497	Housing area	64.074	0.455
R^2^	0.681		R^2^	0.692	
F	43.855		F	45.954	
P	<0.001		P	<0.001	

### Characteristics of Urban Residential GHG Emissions

**Figure 5:**
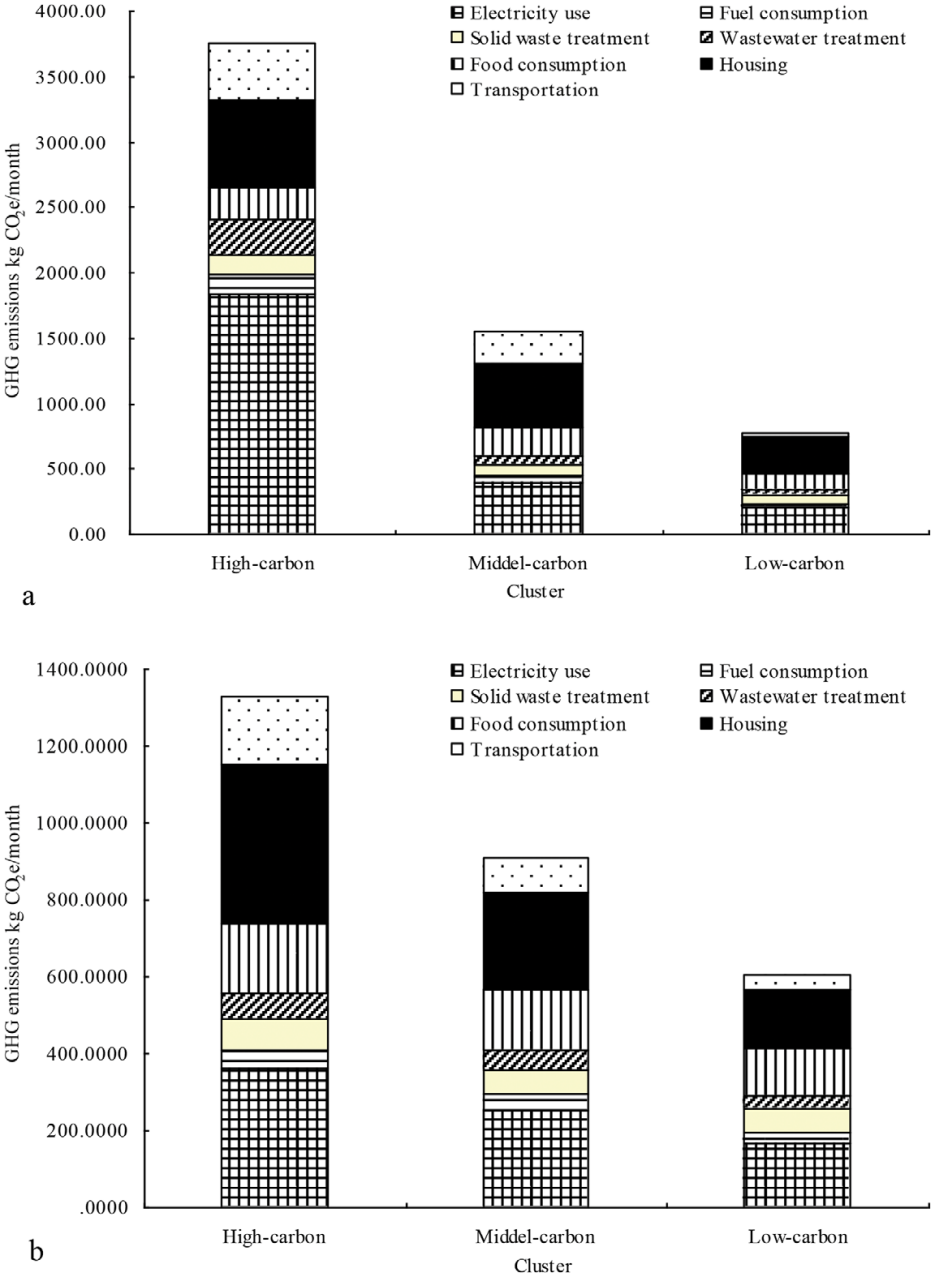
GHG emissions from residential consumptions in the high, medium and low carbon household (a) and community (b) of Xiamen City. Note: a represents households; b represents communities.

At the household scale, taking housing area, household size, building age, household income, and GHG emissions per household and per capita as the analysis variables, the 714 surveyed households are categorized into three groups (low, medium and high GHG emission households) using K-means cluster analysis (see Table 7). The final cluster centers are computed as the mean for each variable within each final cluster and reflected the typical characteristics of the three household categories. A high GHG emission household is always characterized as consisting of 4 persons with more than 150 m^2^ of housing area, living in a building constructed after 2000, and with a monthly household income of 10,000–15,000 yuan. A low GHG emission household is characterized as 3–4 persons with about 80–90 m^2^ of housing area, living in a building constructed in the 1990s, and with a monthly household income of 6000 yuan. High GHG emissions households emit 4.86 times more than low GHG emissions households. Comparing low and high GHG emissions households, the increase in GHG emissions from electricity use per household is the most significant, followed by increases from housing, transportation and wastewater treatment. Increases are also observed in the other three categories of residential consumption, but the growth rates are very small (see figure 5a).

**Table 7:** K-Means cluster analysis of urban residential GHG emissions.

Analysisvariables	Final cluster centers			ANOVA	
Household (n)	Low (497)	Medium (206)	High (11)	F*	P
Household size	3.4	3.77	4	8.714	<0.001
Housing area	2.79	4.23	5.36	140.285	<0.001
Building age	2.72	3.24	3.45	32.565	<0.001
Household income	2.2	3.04	3.27	63.282	<0.001
Per household	770.60	1553.25	3750.46	1133.478	<0.001
Per capita	251.79	460.39	991.28	244.855	<0.001
Community (n)	Low (10)	Medium (24)	High (10)		
Building Height	2.44	3.18	4.00	13.810	<0.001
Average housing area	1.00	1.33	1.90	12.340	<0.001
Per household	701.04	986.03	1466.79	99.600	<0.001
Per capita	223.27	302.78	454.69	59.370	<0.001

Notes:

* F = variance of the group means/mean of the within group variances. The bigger the F value is, the more significantly different the sample groups are.

At community scale, taking average housing area, building height, and GHG emissions per household and per capita as the analysis variables, the 44 surveyed communities are categorized into low, medium, and high GHG emission communities (see Table 7). The final cluster centers show that high GHG emission communities are usually characterized by an average housing area of about 120 m^2^ and buildings usually with eight floors or more. Communities with a lower level of GHG emissions are characterized by an average housing area of about 70–80 m^2^ and buildings with seven floors or fewer. The difference between low and high GHG emissions communities is less than at the household level, but high GHG emissions communities emitt 2.09 times as much as low GHG emissions communities. From low to high GHG emissions communities, the increase in emissions from housing is the most significant, followed by electricity use and transportation. An increase is also observed in the other four residential consumption categories, but the growth rates are very small (see figure 5b).

## Discussion

### Characterizing GHG Emissions from Urban Residential Consumption

The lifestyles of city residents are influenced by physical, social, economic factors, as well as the cultural background which affect GHG emissions in various ways. A bottom-up social survey can directly connect lifestyle factors to the GHG emissions from residential consumption and provide potential breakthrough points for carbon reduction policymaking. For example, housing area was the main influencing factor of residential GHG emissions at the household scale in Xiamen City. If other factors remained constant, larger housing area would result in larger GHG emissions, so policies to reduce housing area per urban household would be an effective measure to control residential GHG emissions for Xiamen City. Currently low-storey buildings are being rapidly replaced by high-rise buildings in Chinese cities to increase compactness [37] and this is also believed to have the co-benefit of reducing GHG emissions [38]. However, our results show that high-storey residential buildings and spacious housing both tend to increase GHG emissions from urban residential consumption. It is hard to develop a low-carbon city simply by increasing the compactness of residential buildings. Effective carbon reduction policies must therefore consider other ways to reduce emissions from residential consumption, as will be discussed below.

Another advantage of the survey based approach is that it offers the possibility to further break down the underlying factors. Household size is widely recognized as a major factor influencing residential GHG emissions[10], [17], [39]–[41], and larger households tend to be more efficient in terms of per capita energy use [10], [40], [42]. Our study did find that residential GHG emissions per capita tended to decrease with increasing household size, but only to an optimum household size of four persons. A four-member family could be comprised of, for example, a middle-aged couple with two children, a middle-aged couple with one child and an elderly parent, or an elderly couple living with a child and his/her spouse. Family composition may be a key underlying factor in determining residential GHG emissions and merit further study.

Residential consumption will play an increasingly important role in future to shape China’s energy demand and GHG emissions. It is necessary to understand the tendencies of Chinese urban lifestyles to achieve low-carbon city development. In our study, the influential factors of residential GHG emissions presented similar trends from low to high GHG emissions households and communities (see Table 7). This GHG emissions gradient existing among present households and communities can provide valuable information on the likely future changes in Chinese urban residential consumption. Currently, most urban households and communities in Xiamen are low or medium carbon emitters (see Table 7). Future urbanization and socioeconomic development is likely to result in increasing income levels, housing renovation, an increase in housing area and the replacing of low-storey buildings with high-rise apartment blocks. As a result, the proportion of low GHG emissions households and communities will gradually reduce while high-carbon households and communities is likely to increase rapidly. At the same time, the composition of residential GHG emissions will change, and GHG emissions from housing and transportation may grow significantly.

### Policy Making Toward a Low-carbon Urban Consumption Pattern

Jones and Kammen argued that realizing GHG emissions reduction required behavior change at the household level through personalized feedback [14]. This makes theoretical sense, because the most effective measure to reduce GHG emissions from household consumption is to cut unnecessary material or energy use directly. However, our results suggest that from a lifecycle perspective, the largest carbon reduction potentials are beyond the control of individual consumers. For example the majority of urban residential GHG emissions in Xiamen City are mainly derived from urban (17%) and regional and national (70%) economic activity. As a result, policy measures such as extending building lifespan and the recycling of building wastes could contribute more significantly to GHG emissions reduction than simply targeting individual consumer choices alone. The percentage of clean primary energy in the total energy use is only 3% in China [43]. Adjusting the composition of primary energy to produce electricity may have a greater potential for carbon reduction than simply reducing household electricity use.

Policymaking for a low-carbon city must therefore adopt a holistic approach in terms of policy scope, priority and timing of implementation. Taking Xiamen City for example, the policy scope should cover the entire path of primary energy or materials to end-users, including household behavior and urban, regional and national activity. Specific policies should include: promoting energy saving appliances and greater use of public transportation at the household scale, promoting low-carbon techniques of pollution control, such as clean coal technology, catalytic combustion technoloy, increasing the proportion of food that is locally sourced at the city scale, adjusting the primary energy mix for electricity production and developing green building materials and technologies at the regional or national scale.

Policy priority should be given to residential consumption which results in the greatest GHG emissions, including housing, electricity use, food consumption and transportation. Further studies will be needed to quantify the carbon reduction potentials in each consumption category given current technology and to assess practical feasibility. Due to the large disparity in GHG emissions profile between different households and communities, high-carbon households and communities should be the target of policies to promote lifestyle adjustments.

Timing of policy implementation should be based on predictable changes in urban lifestyle and focus on residential consumption which is expected to increase significantly in the near future. Green building materials and technologies to reduce GHG emissions from housing construction are the most urgent, followed by promoting the proportion of clean energy in electricity production, increasing the efficiency of household electricity use, and encouraging the use of public transportation.

### Conclusions

As cities become the primary habitat of human beings, GHG emissions from urban residential consumption and the role of urban lifestyle has become increasingly significant. We present a survey-based GHG emissions accounting methodology for urban residential consumption and apply it in Xiamen City, China. According to our results, reducing the GHG emissions from urban residential consumption is often beyond the control of individual consumers. Housing, electricity use and food consumption whose GHG emissions are from regional and national economic activities (PR-sourced) and wastewater treatment and solid waste treatment whose GHG emissions are from urban economic activities (PU sourced) accounted for about 70% and 17% of total residential GHG emissions in Xiamen City respectively. The entire energy or materials pathway to the end-users should be included in the policymaking scope. A large disparity in carbon profile between different households, with the high carbon households emitting about five times as much GHG as low carbon households. High carbon communities emit about twice as much GHG as low carbon communities. Residential consumptions which resulted in the majority of GHG emissions and which would likely increase significantly in the near future including housing, electricity use, and transportation, should be the key points for policymaking of low-carbon urban residential consumption in China. The survey-based GHG emissions accounting method of household consumption developed in this study can be readily applied to other cities. It provides a useful tool to understand and profile residential groups, and makes it possible to design tailored and targeted policies for GHG emissions reduction.
